# *Ralstonia picketti* neonatal sepsis: a case report

**DOI:** 10.1186/s13104-016-2347-1

**Published:** 2017-01-07

**Authors:** Deepak Sharma, Pradeep Sharma, Priyanka Soni, Basudev Gupta

**Affiliations:** 1 NEOCLINIC, TN Mishra Marg, Everest Vihar, Nirman Nagar, Jaipur, Rajasthan India; 2 Department of Medicine, Mahatma Gandhi Medical College, Jaipur, Rajasthan India; 3 Department of Microbiology, J.L.N Medical College, Ajmer, Rajasthan India; 4 Department of Pediatrics, Civil Hospital, Palwal, Haryana India

**Keywords:** *Ralstonia picketti*, Neonate, Sepsis

## Abstract

**Background:**

Ralstonia genus are gram negative bacillus and includes four bacteria namely *Ralstonia picketti*, *Ralstonia Solanacearum*, *Ralstonia insidiosa* and *Ralstonia mannitolilytica*. These are opportunistic pathogens and cause infections in immunocompromised host. The sources of infection are usually contaminated solutions and water. The majority of the reported cases are caused by *R. picketti*. It is very rare cause of neonatal sepsis with less than twenty cases reported in literature till date.

**Case presentation:**

A late preterm male infant, Indian race was admitted to the neonatal intensive care unit for respiratory distress developing soon after birth. The infant was managed with respiratory support and gradually infant improved and diagnosis of transient tachypnea of newborn was made. At age of 84 h of postnatal life, the infant developed features of neonatal sepsis and investigations were suggestive of sepsis. The infant was started on intravenous antibiotic, multiple vasopressors and steroids. The blood culture showed growth of multi-drug resistant *R. picketti*. The antibiotics were changed as per sensitivity pattern and infant was discharged in good condition and was accepting breast feeding at the time of discharge. There was also no other case of *R. picketti* in the nursery during the same time period.

**Conclusion:**

*Ralstonia picketti* is an uncommon cause of neonatal sepsis and usually source of infection are contaminated solutions and medical products. The management involves early detection, treatment with appropriate antibiotics and doing surveillance culture to identify the possible source of infection.

## Introduction

Ralstonia was first isolated in 1973 and was included in the genus *Pseudomonas* spp. Subsequently, this microorganism was reclassified into the genus Burkholderia (*Burkholderia picketti* and *Burkholderia solanacearum*). The genus Ralstonia was named separately in 1995 by Yabuuchi et al. [1]. Ralstonia are aerobic gram-negative bacillus, non-fermentative rods found in water and soil. They have low-virulence and are associated with nosocomial sepsis by health care personal. Ralstonia genus includes four bacteria namely *Ralstonia picketti*, *Ralstonia Solanacearum*, *Ralstonia insidiosa* [2] and *Ralstonia mannitolilytica* [3]. The majority of the reported cases are caused by *R. picketti*, but there are very few case report of *R. picketti* infection in neonate. We here report a case report of *R. picketti* in neonate leading to late onset neonatal sepsis. The organism was multi-drug resistant and the infant was treated with appropriate antibiotics and was discharged in good condition.

## Case presentation

A late preterm male infant (34 weeks), Indian race having birth weight of 2350 g (appropriate for gestational age) was referred for admission to the neonatal intensive care unit (NICU) for respiratory distress developing soon after birth. The infant was 2 h old at the time of admission to the NICU. The infant was born to 24-year old primi-gravida mother via normal vaginal delivery. The cause of prematurity was preterm labour and there were no features of chorioamnionitis in the mother. The clinical examination of the newborn at the time of admission showed respiratory rate of 68/min, heart rate of 154 beats/min, capillary refill time of 2 s, audible grunting with subcostal and intercostal retraction, nasal flaring, normal heart sounds and Silverman Anderson Score of 5/10. The infant was started on nasal Bubble CPAP (Continuous positive airway pressure) in view of respiratory distress and was evaluated with chest radiograph, which had features of transient tachypnea of newborn (TTNB). The infant was started on intravenous fluid and was investigated with sepsis screen. There was gradual improvement in respiratory distress in the next 48 h, therefore nasal CPAP was removed but the infant had minimal oxygen requirement for which oxygen was given using nasal prong. The initial blood investigations [total leucocyte count, absolute neutrophil count, C-Reactive protein (CRP)] were not suggestive of any sepsis therefore antibiotics were not started. The infant was started on tube feeds and the feeds were gradually increased. At age of 84 h of postnatal life, the infant developed features of neonatal sepsis (mottling, decreased activity, abdominal distension, cold and dusky periphery, off colour, delayed capillary refill time (>4 s), abdominal distension, feed intolerance with altered pre-feed aspirate, low volume pulses, tachycardia (180–184 beats/min), low blood pressure (mean blood pressure 24 mmHg) and tachypnea (respiratory rate >60/min). The infant was again evaluated with total leucocyte count, absolute neutrophil count, CRP, blood culture and chest radiograph and was started on antibiotic (piperacillin–tazobactum) in suspicion of late onset sepsis (LOS). The infant hypotension was managed with multiple vasopressors and steroids. The investigations showed total leucocyte count of 3200/microliter (leucopenia), absolute neutrophil count 876/microliter (moderate neutropenia), platelet count of 25,000/microliter (severe thrombocytopenia), Immature neutrophil/Total neutrophil (I/T) ratio of 0.42, toxic granulations on peripheral smear and very high CRP (220 mg/l) (normal value <10 mg/l). Blood gas analysis showed mixed acidosis with pH of 7.242, Pco2 46 mmHg, bicarbonate 10.2 mmol/l and base excess of −15 mmol/l. The chest radiograph showed pneumonia of right lung and the infant required invasive ventilation because of impending respiratory failure for next 48 h. The infant also received packed cell and platelet transfusion for anemia and severe thrombocytopenia. The cerebrospinal fluid (CSF) analysis was done in view of LOS, which was suggestive of normal study (CSF sugar 84 mg/dl against blood sugar of 110 mg/dl, CSF protein being 104 mg/dl and cell count showed 02 cells/microliter with 100% cells being lymphocytes). The blood culture was send from two different site using strict aseptic precaution with three swab technique, showed growth of *R. picketti*. The sensitivity pattern of the organism showed sensitivity to Tetracycline and Tigecycline, resistance to Cefoperazone sulbactum, Ciprofloxacin, Colistin, Amikacin, Amoxyclav, Ampicillin, Cefazolin, Cefepime, Cefotaxime, Ceftazidime, Cefixime, Ertapenem, Gentamicin, Imipenem, Levofloxacin, Meropenam, Piperacillin + Tazobactum, Piperacillin, Ticarcillin, Trimethoprim/sulfamethoxazole and Tobramycin. The CSF culture was not suggestive of any bacterial growth. The antibiotics were upgraded from piperacillin–tazobactum to Tigecycline as per the sensitivity pattern. The clinical condition of the infant gradually improved and vasopressors were subsequently tapered and stopped. The repeat blood culture sent after day 14 of Tigecycline showed no growth. The infant was discharged in good condition and was accepting breast feeding at the time of discharge. The surveillance cultures were sent (hospital water supplies, saline solutions, medicine vials and respiratory therapy equipments) to find the source of infection but we were unable to find the source of infection in the index case. There was also no case of Ralstonia in the nursery during the same time period.

## Conclusions

Ralstonia are gram-negative bacillus and rarely causes primary bacteremia and the infection is usually secondary to contaminated medical products, fluids and/or medications. *R. picketti* (formerly known as *Pseudomonas picketti* and *Burkholderia picketti*) is most commonly reported pathogen for sepsis and has been isolated from various clinical specimens like blood, urine and cerebrospinal fluid [4], from water including municipal drinking water supplies [5, 6], bottled water [7], dental water supplies [8, 9], hospital water supplies [10], space shuttle water systems, standard purified water, laboratory based high-purity water systems and industrial Ultra-pure/High Purity water, from plastic industrial water piping, medicine vials [11], contaminated saline solution [12], from respiratory therapy solutions and wound irrigation systems [13], and blood culture bottles [14]. This organism is not considered as a major pathogen and therefore it is not routinely looked for in hospital analysis. Ralstonia is an opportunistic human pathogen and seen in immunocompromised condition, as seen in the present case. The premature neonates are in an immunocompromised state making them prone to develop this infection. The recent studies have shown that Ralstonia is a growing opportunistic pathogen whole over the world [15].

*Ralstonia picketti* is an inhabitant of environment, is not considered a part of normal human flora and has been reported to cause nosocomial outbreak secondary to contaminated fluids [16]. Their transmission usually requires human contact with heavily contaminated medical devices or substances encountered in the hospital settings. The exact mode of transmission is not known but it likely involves exposure to contaminated medical devices. As *R. picketti* is one of the uncommon cause of infection in humans, very little is known about associated virulence factors [17].

The microbiological properties show no distinct appearance on blood agar and they are non-lactose fermenter on Mac Conkey Agar. The biochemical properties show variable growth at 42 °C, gas from nitrate and gelatin liquefaction, oxidation of lactose, positive for nitrate reduction, urea hydrolysis, xylose oxidation and glucose oxidation and negative for arginine hydrolysis, Lysine decarboxylase and oxidation of mannitol [4].

The treatment of *Ralstonia* spp. sepsis is difficult and challenging because this pathogen is resistant to many commonly used antibiotics, like β-lactams and aminoglycosides [18]. In the index case, the pathogen was only sensitive to Tetracycline and Tigecycline. We used Tigecycline, a tetracycline derivative as baby condition was critically sick and when we assessed risk benefit ratio we decided to start Tigecycline and baby improved after the change of antibiotics. There is lack of validated susceptibility testing that poses a substantial risk of erroneous interpretations. There are no definitive guidelines and the reliable therapeutic data is limited as *R. picketti* is one of the rarely isolated organism from human specimens. Ryan et al. in their study reported that numerous strains of *R. picketti* and *R. insidiosa* were highly resistant to the aminoglycoside (gentamicin) and the ß-lactam antibiotic aztreonam and were variably resistant to the Ticarcillin-clavulanic acid. The best antibiotic in the study were sulfamethoxazole/trimethoprim and the fluoroquinolone (ciprofloxacin) [19].

There are no recommended vaccination or prophylaxis protocols for Ralstonia because of the rarity of the infection despite the ubiquity of the organism. Hospital acquired infections can be controlled through the use of appropriate sterile technique and implementation of strict protocols for sterilization and disinfection of hospital supplies [15].

Kimura et al. conducted case–control study to determine risk factors for infection following outbreak of *Ralstonia picketti* bacteremia in nursery. There were 18 patients with 19 episode of infection and all cases were within 1 month. There was no neonatal death secondary to bacteremia because of low virulence of the bacteria. The source of this outbreak was contaminated heparin flush and the epidemic ended with discontinuation of it [20].

Vitaliti et al. reported a case of neonatal sepsis caused by *R. picketti* in a 26-week infant. The infant had Ralstonia sepsis at 25 days of postnatal life in form of apnea and bradycardia requiring mechanical ventilation. The infant died secondary to disseminated intravascular coagulation and fatal multiorgan failure [21].

Bonatti et al. reported a case of *R. picketti* in 29-week preterm infant. The infant had post-hemorrhagic hydrocephalus that was managed with ventriculo–peritoneal shunt system and there was isolation of *R. picketti* from the cerebrospinal fluid. This infection was thought secondary to the shunt and the preterm was discharged in well condition [22].

Forgie et al. reported a case of neonate who was operated for hypoplastic left heart syndrome at age of six day of postnatal life. The neonate received extracorporeal membrane oxygenation (ECMO) post-operatively and neonate developed *R. picketti* sepsis secondary to ECMO circuit. The neonate was treated with ciprofloxacin for 14 days and was discharged successfully [23].

Pandey et al. from India reported three case of *R. picketti* bacteremia in adult intensive care unit. The two patients succumbed to death were case of leukemia and were on immunosuppressive therapy and third case of thalassemia, even though on immunosuppressive medications was discharged successfully [24].

There is need for vigilance over the infection caused by Ralstonia especially in intensive care unit where they are more prone to cause sepsis. The role of routine antibiotic prophylaxis for Ralstonia is not recommended by us as the infection is very rare and widespread use of the antibiotics may lead to multidrug resistance in this organism. The health care person should have extra vigilance of other patients if a single case of Ralstonia is found (e.g. cause could be a contaminated saline solution batch or medicine vial) and should test specifically for *R. picketti* if multiple cases of sepsis suddenly present. There is recently an increase in the incidence of Ralstonia sepsis and this may be a time for physicians to consider whether changes are required to the current norms in prophylaxis, detection and/or monitoring.

*Ralstonia picketti* is not recognized as major pathogen and is an uncommon cause of neonatal sepsis. The source of infection is usually contaminated solutions and medical products. The management involves early detection, treatment with appropriate antibiotics and doing surveillance culture of the possible sources to identify the correct source.

## Abbreviations

Bubble CPAP: bubble continuous positive airway pressure
CSF: cerebrospinal fluid
ECMO: extracorporeal membrane oxygenation
LOS: late onset sepsis
NICU: neonatal intensive care unit
TTNB: transient tachypnea of newborn
